# Supplementary material for: Plasmodium RON11 triggers biogenesis of the merozoite rhoptry pair and is essential for erythrocyte invasion
Source: PLoS Biol. 2024 Sep 18;22(9):e3002801. doi: 10.1371/journal.pbio.3002801 (PMC11441699; doi:10.1371/journal.pbio.3002801)
Supplement: S1 Table — (DOCX) [file pbio.3002801.s003.docx]

**S1 Table.** List of primers used in the study to generate the cell lines RON11^apt^.

| **Amplicon** | **Primer** | **Sequence (5’ – 3’)** |
| --- | --- | --- |
| RON11-Cterm  integration | P1 | TGTTTAGGAATTTTTGGAGGTATTTGATAG |
| Apt integration | P2 | CTAGACTAGGTTCCAAGATCTCCC |
| RON11-Cterm | P3 | ATTGTGTATCCCGATATCGATGTTAATAGTTTAAAAAAAATACTAACAGAAGTAAGATG |
|  | P4 | CGTCATAAGGGTATCCGGAGACGTCTTTCGAATCAGATGCTGACTGTAAATAAG |
| RON11-3’UTR | P5 | CCAATGGCCCCTTTCCGGGCGCGCCCTTTTTTGTTTGAAATTGGCAAAAATATTAGTAG |
|  | P6 | ACTATTAACATCGATATCGGGATACACAATAGAATATTAATTAAGTGTATTATTAAGTG |
| RON11 gRNA | P7 | CATATTAAGTATATAATATTGATATGGATGTCGAAAGAAAGTTTTAGAGCTAGAAATAGC |
