# Supplementary figures and images for: Plasmodium RON11 triggers biogenesis of the merozoite rhoptry pair and is essential for erythrocyte invasion
Source: PLoS Biol. 2024 Sep 18;22(9):e3002801. doi: 10.1371/journal.pbio.3002801 (PMC11441699; doi:10.1371/journal.pbio.3002801)

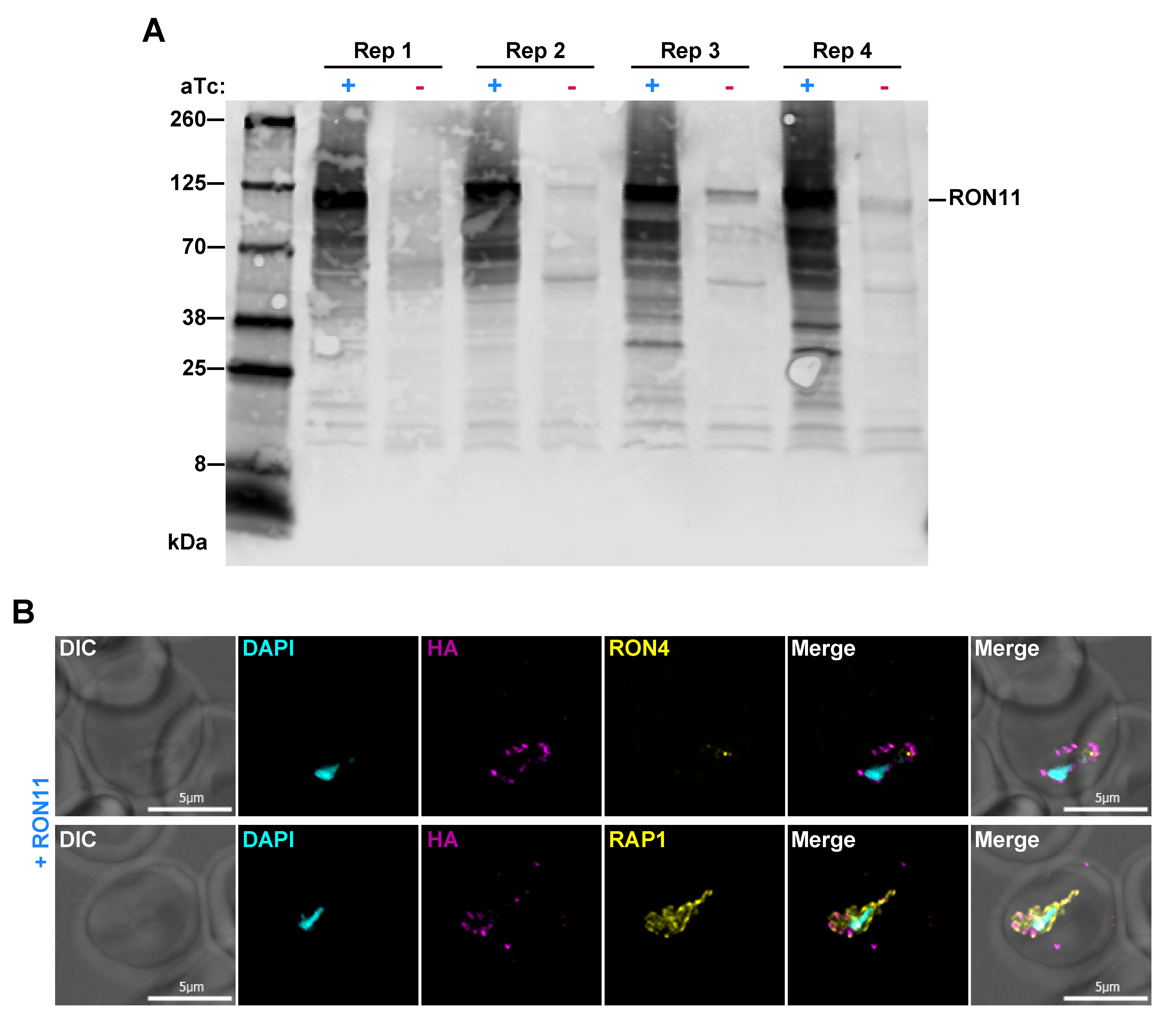

Supplement: S1 Fig — (A) Overexposed western blot of the knockdown of RON11 shown in Fig 1C. Lysates were collected from E64-arrested RON11apt parasites in the presence or absence of aTc. Samples were probed with antibodies against the HA tag. The protein marker sizes are shown on the left. Blot shows 4 biological replicates. (B) IFAs showing the localization of RON11 in RON11apt rings with respect to the rhoptry markers RON4 and RAP1. Synchronous parasites were fixed with PFA and stained with specific antibodies. Images from left to right are DIC, DAPI (nucleus, cyan), anti-HA (RON11, magenta), anti-RON4 or RAP1 (yellow), and fluorescence merge. Z stack images were deconvolved and projected as a combined single image. Representative images of 2 biological replicates. (TIF) [file pbio.3002801.s005.tif]

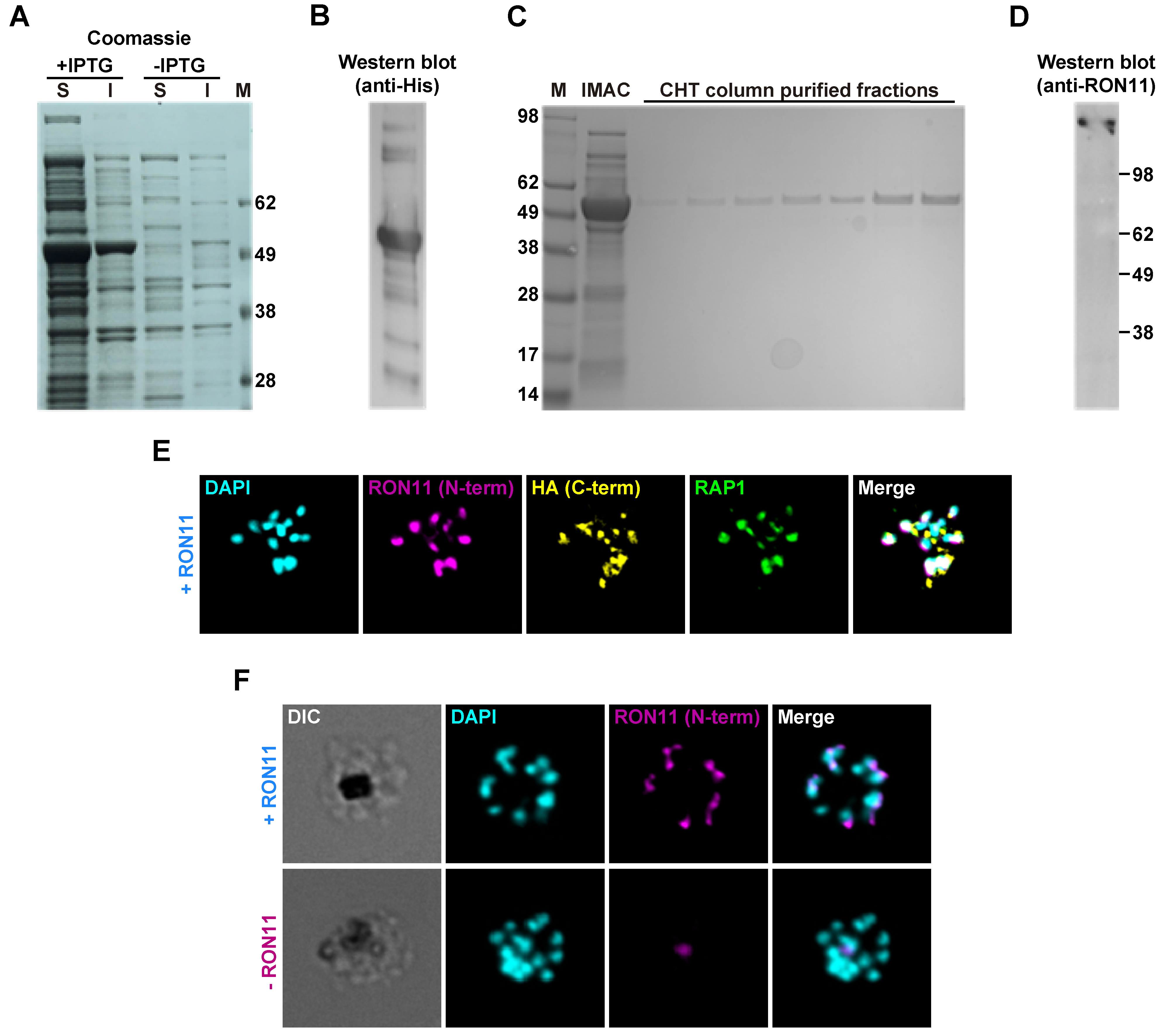

Supplement: S2 Fig — (A) Expression of rMBP-RON11 (aa 32–442) protein in E.coli. Soluble (S) and insoluble (I) fractions from cell lysates without and without IPTG induction. M; Marker. (B) Western blot analysis of the soluble fraction using anti-His antibody and detected using anti-mouse HRP conjugated secondary antibody. (C) rMBP-RON11 following IMAC purification and subsequent clean-up using CHT column. (D) Western blot analysis of P. falciparum schizont lysates using anti-RON11 antibody. (E) IFAs showing the localization of RON11 in RON11apt mature schizonts. Images from left to right are DAPI (nucleus, cyan), anti-RON11 (magenta), anti-HA (yellow), anti-RAP1 (green), and fluorescence merge. (F) IFAs showing the knockdown of RON11 in RON11apt mature schizonts. Images from left to right are DIC, DAPI (nucleus, cyan), anti-RON11 (magenta), and fluorescence merge. Z stack images were deconvolved and projected as a combined single image. (TIF) [file pbio.3002801.s006.tif]

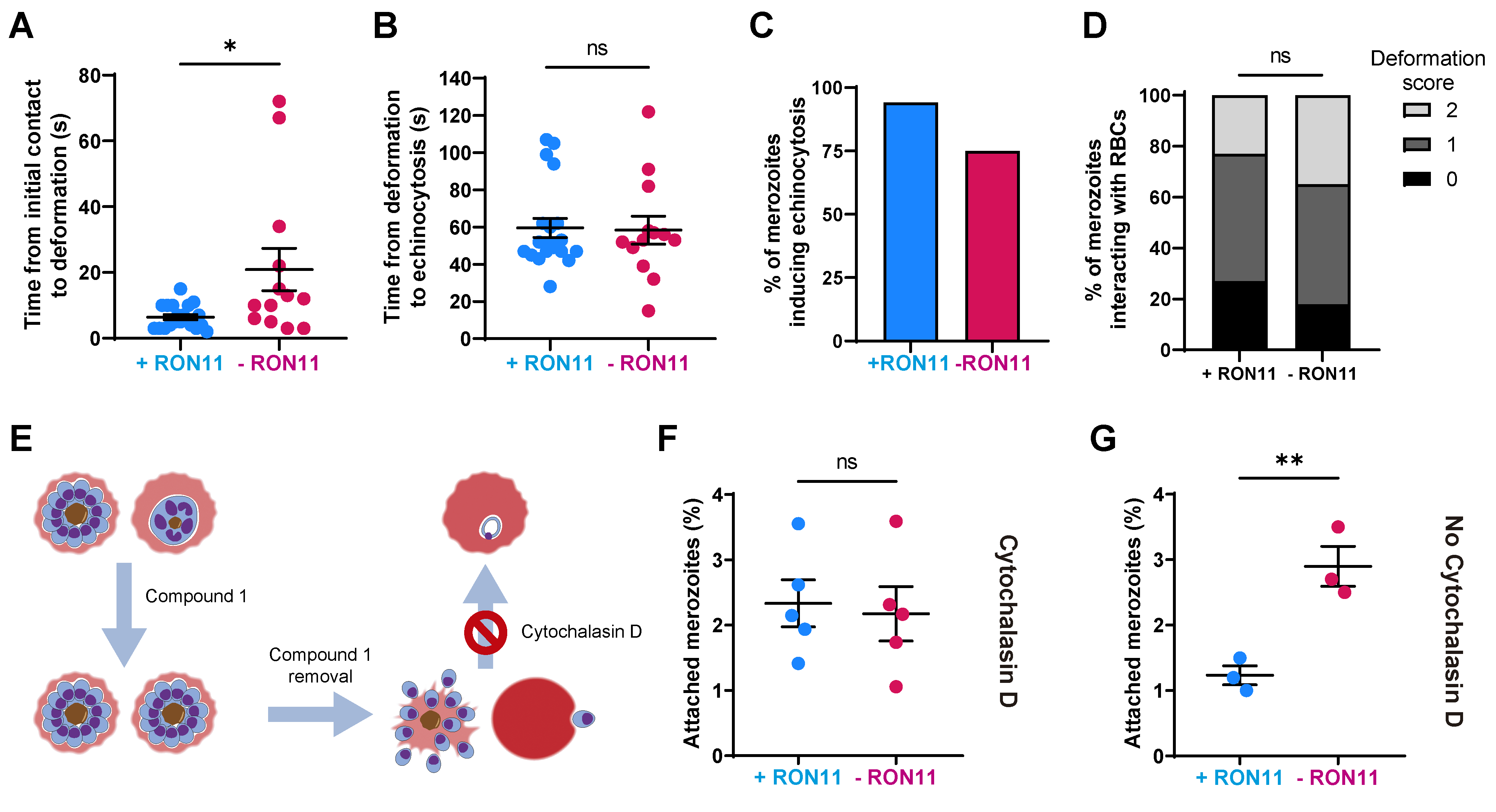

Supplement: S3 Fig — (A) Duration between first contact of merozoites with RBCs and observation of RBC deformation (n = 2 biological replicates, 20 merozoites for +RON11 and 13 for -RON11; error bars = SEM; *p < 0.05 by unpaired two-tailed t test; the underlying data can be found in S1 Data). (B) Duration between merozoite first merozoite-induced RBC deformation and echinocytosis (n = 2 biological replicates, 20 merozoites for +RON11 and 13 for -RON11; error bars = SEM; ns = non-significant by unpaired two-tailed t test; the underlying data can be found in S1 Data). (C) Quantification of RBCs showing echinocytosis after merozoite-induced deformation (n = 3 biological replicates, 34 merozoites for +RON11 and 44 for -RON11; the underlying data can be found in S1 Data). (D) Deformation scores based on the strength of merozoite-RBC interaction. 0 = no deformation; 1 = shallow indentation/membrane pinching; 2 = deeper indentation to the side of RBC/intermediate level of host cell membrane pinching around the parasite. (n = 3 biological replicates, 44 merozoites for each condition; ns = non-significant by chi-squared test; the underlying data can be found in S1 Data). (E) Schematic of the actin-polymerization inhibitor assay. Schizonts were tightly synchronized incubating for 4 h with the PKG inhibitor, Compound 1. After incubation, schizonts were washed twice and then transferred to fresh red blood cells in the presence of the actin inhibitor, cytochalasin D, for 30 min. (F and G) Quantification of RON11apt merozoites attached to RBCs in the presence or absence of aTc, after incubation with (F) or without (G) cytochalasin D. Parasites were smeared 30 min after Compound 1 removal, stained with Hema 3, and scored by light microscopy. Attached-merozoites were blindly scored and represented as the percentage of events per 100 RBCs (n = 5 and 3 biological replicates, respectively; error bars = SEM; ns = non-significant; **p < 0.01 by unpaired two-tailed t test; the underlying data can be found in [file pbio.3002801.s007.tif]

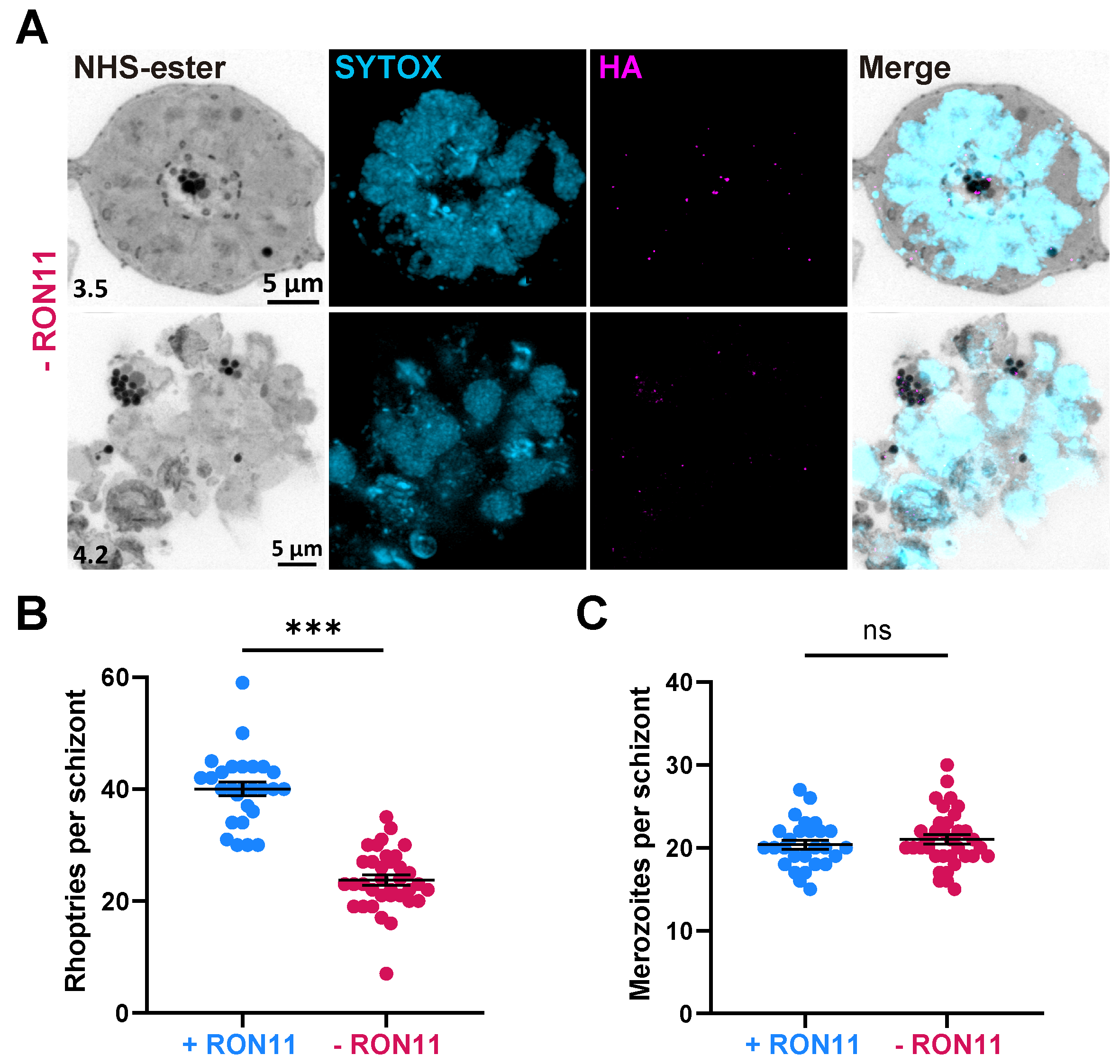

Supplement: S4 Fig — (A) Representative images of RON11apt parasite showing multiple rhoptries accumulating within fully developed schizonts in the absence of aTc. ML10-arrested parasites were expanded by U-ExM, fixed with PFA, and stained with NHS-Ester (grayscale), anti-HA (magenta), and the DNA dye SYTOX (cyan). Selected Z stack images were projected as a combined single image. Number on image = Z-axis thickness of projection in μm. (B and C) Quantification of (B) rhoptries per merozoites and (C) merozoites per schizont in the presence and absence of aTc. Merozoites were scored based on the number of nuclei observed (n = 4 biological replicates, 28 schizonts for +RON11 and 36 for -RON11; error bars = SEM; ns = non-significant; ***p < 0.001 by unpaired two-tailed t test; the underlying data can be found in S1 Data). (TIF) [file pbio.3002801.s008.tif]

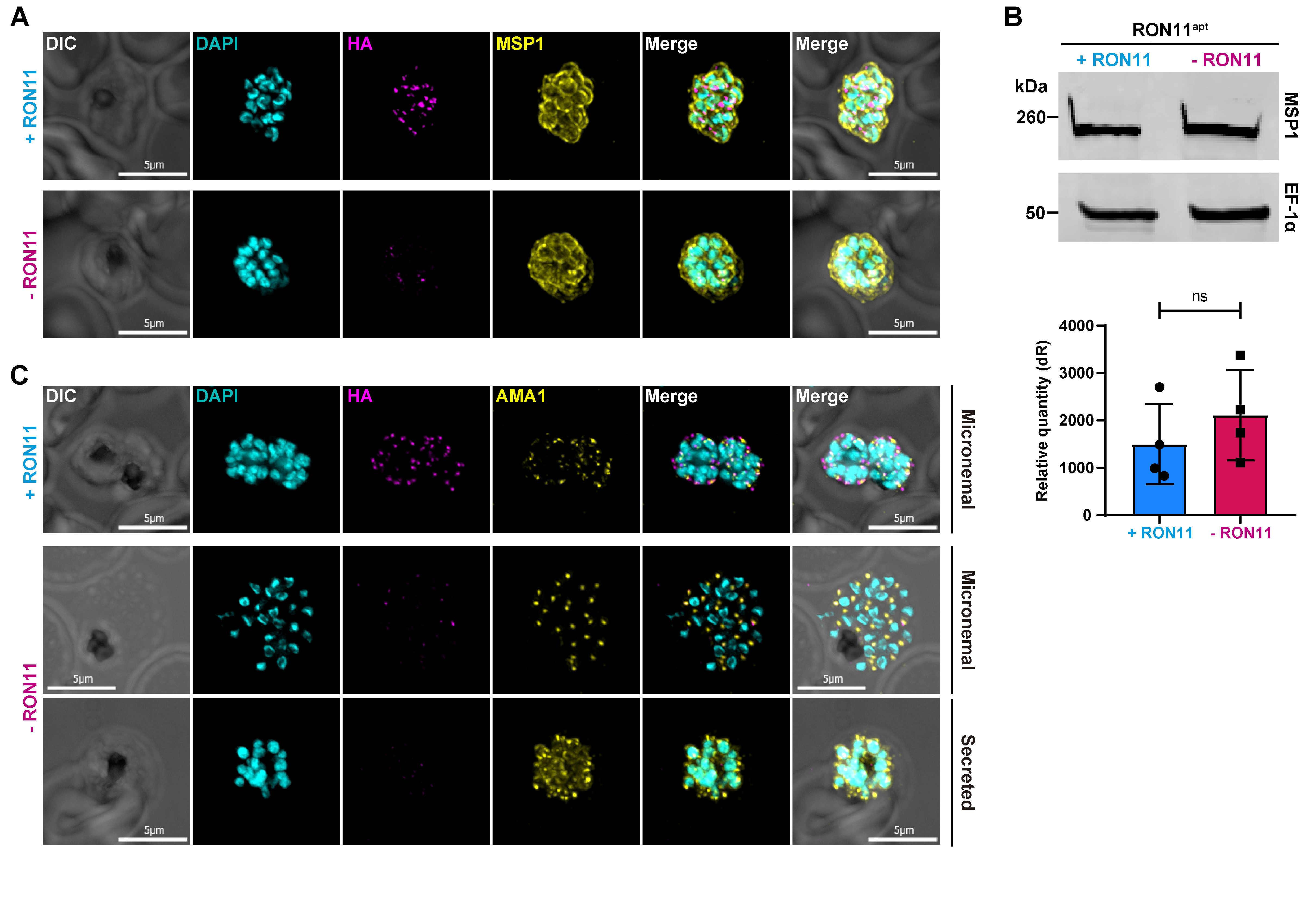

Supplement: S5 Fig — (A) Representative IFAs of RON11apt schizonts showing secretion of MSP1 proteins in the presence or absence of aTc. Images from left to right are phase-contrast, DAPI (nucleus, blue), anti-HA (RON11, green), anti-MSP1 (red), and fluorescence merges. Z stack images were deconvolved and projected as a combined single image. Representative images of 3 biological replicates. (B) (Top) Western blot of parasite lysates isolated from E64-arrested RON11apt parasites in the presence or absence of aTc. Samples were probed with antibodies against MSP1 and EF1α (loading control). The protein marker sizes are shown on the left. Representative blot of 4 biological replicates shown. (Bottom) Quantification of MSP1 in E64-arrested RON11apt parasites in the presence or absence of aTc. Band intensities were normalized to the loading control, EF1α (n = 4 biological replicates; error bars = SD; ns = non-significant by unpaired two-tailed t test; the underlying data can be found in S1 Data). (C) Representative IFAs of RON11apt schizonts showing AMA1 localization in the presence or absence of aTc. Images from left to right are DIC, DAPI (nucleus, blue), anti-HA (RON11, green), anti-AMA1 (red), and fluorescence merges. Z stack images were deconvolved and projected as a combined single image. Representative images of 3 biological replicates. (TIF) [file pbio.3002801.s009.tif]

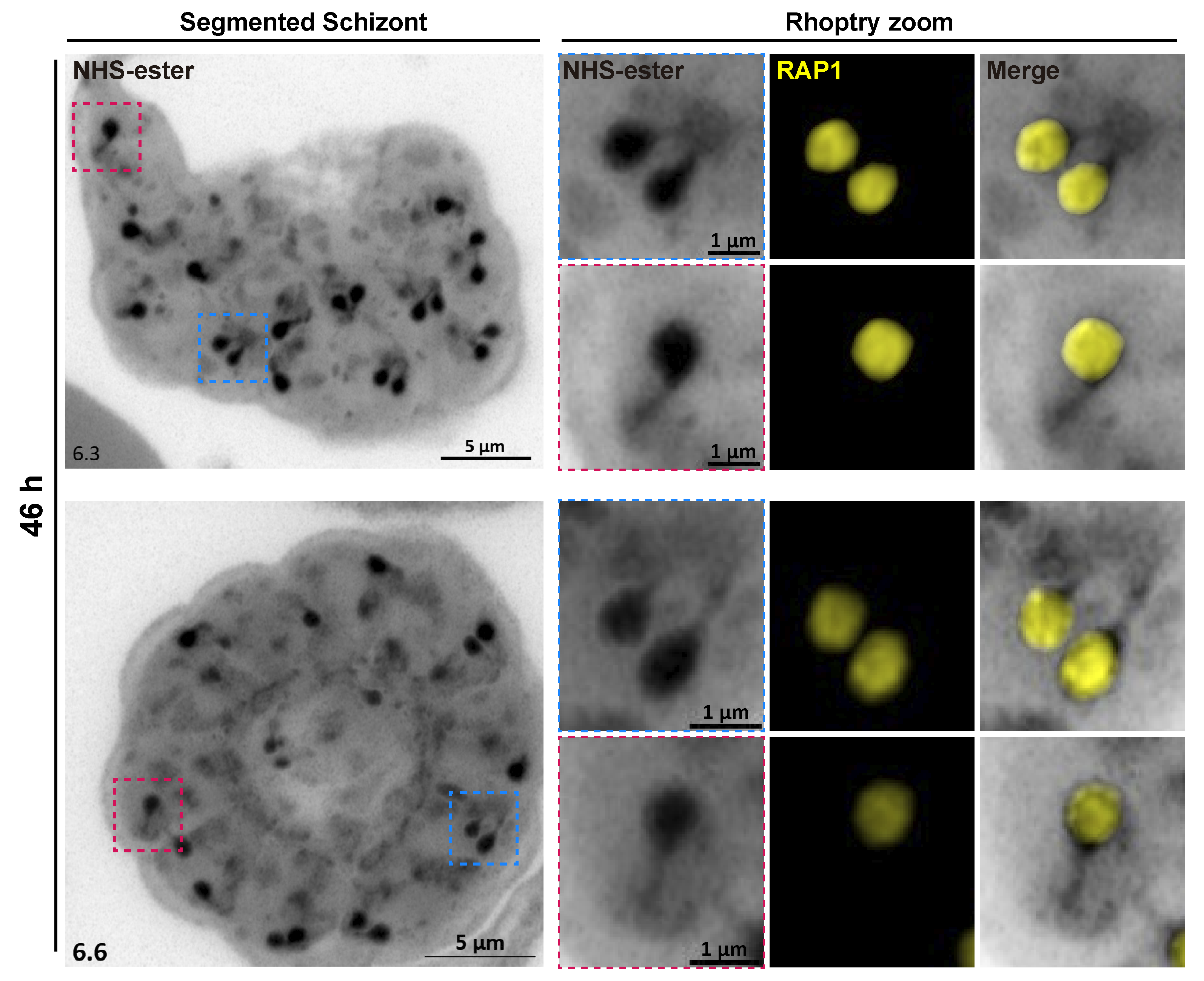

Supplement: S6 Fig — Representative U-ExM images of E64-treated late RON11apt schizonts after supplementing back aTc at 46 hpi. Late-schizont stage parasites were stained with NHS-Ester (grayscale) and anti-RAP1 (yellow). Selected Z stack images were projected as a combined single image. Number on image = Z-axis thickness of projection in μm. (TIF) [file pbio.3002801.s010.tif]

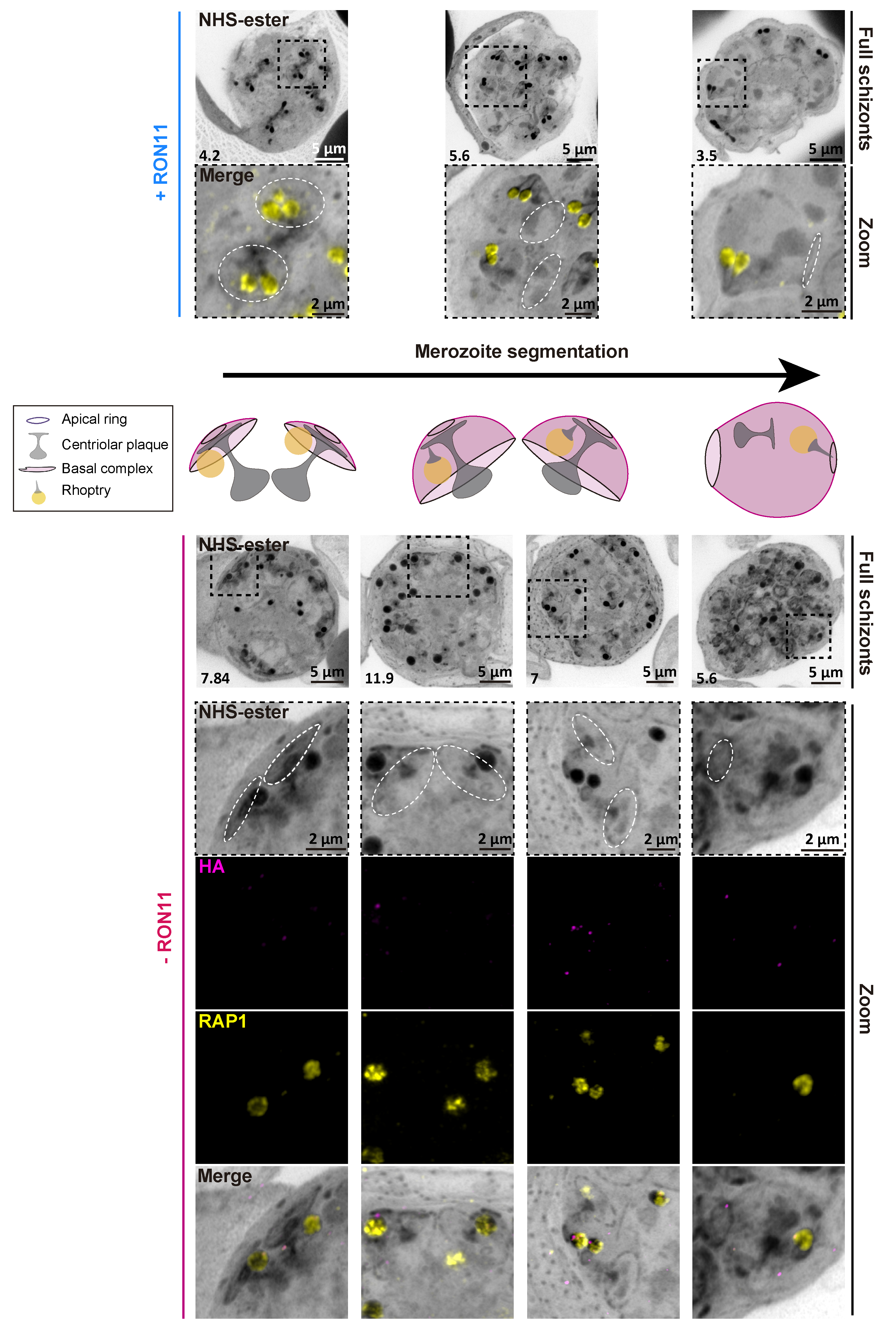

Supplement: S7 Fig — Representative images of different steps of rhoptry biogenesis in RON11apt late schizonts in the presence or absence of aTc. Basal complexes are demarcated by a discontinuous white line. The top panel shows the start of merozoite segmentation by the basal complex and each successive panel shows later stages of segmentation. Late-schizont parasites were expanded by U-ExM, fixed with PFA, and stained with NHS-Ester (grayscale), anti-HA (magenta), and anti-RAP1 (yellow). Selected Z stack images were projected as a combined single image. Number on image = Z-axis thickness of projection in μm. (TIF) [file pbio.3002801.s011.tif]
