# Supplementary material for: Plasmodium RON11 triggers biogenesis of the merozoite rhoptry pair and is essential for erythrocyte invasion
Source: PLoS Biol. 2024 Sep 18;22(9):e3002801. doi: 10.1371/journal.pbio.3002801 (PMC11441699; doi:10.1371/journal.pbio.3002801)

ORIGINAL BLOTS

Figure 1B

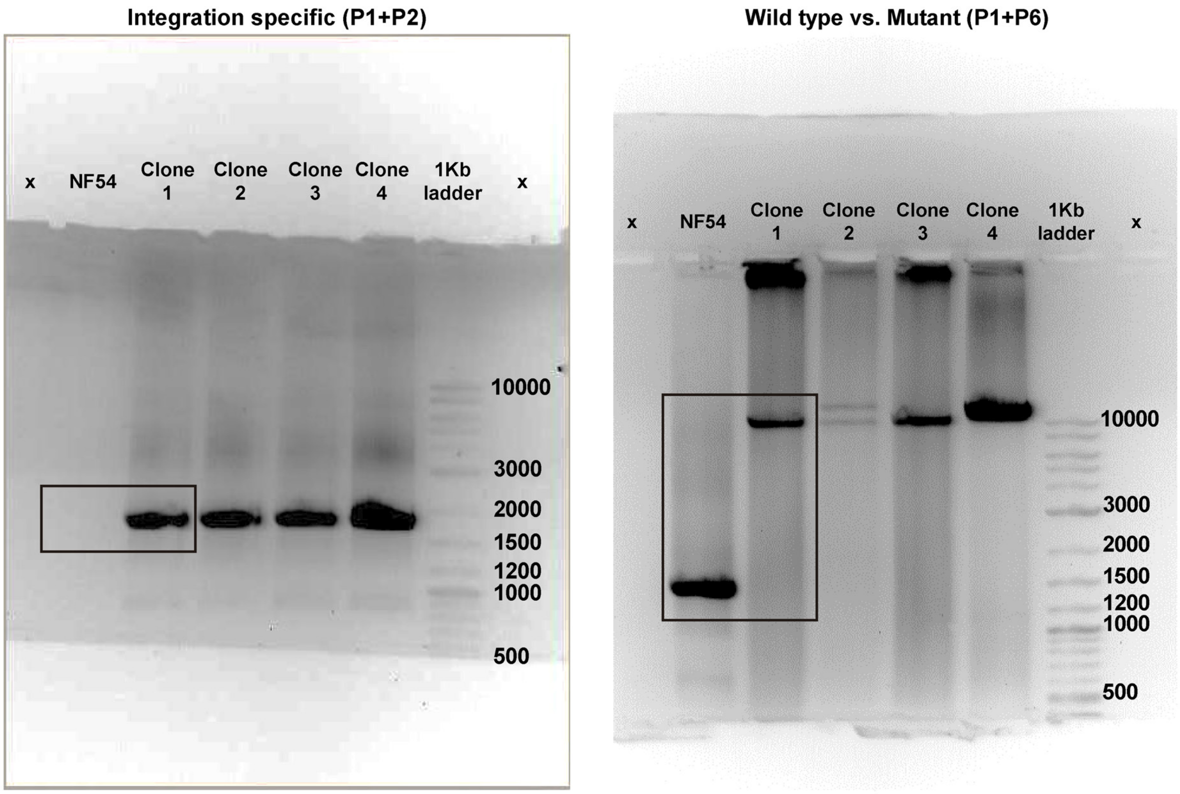

Figure 1C

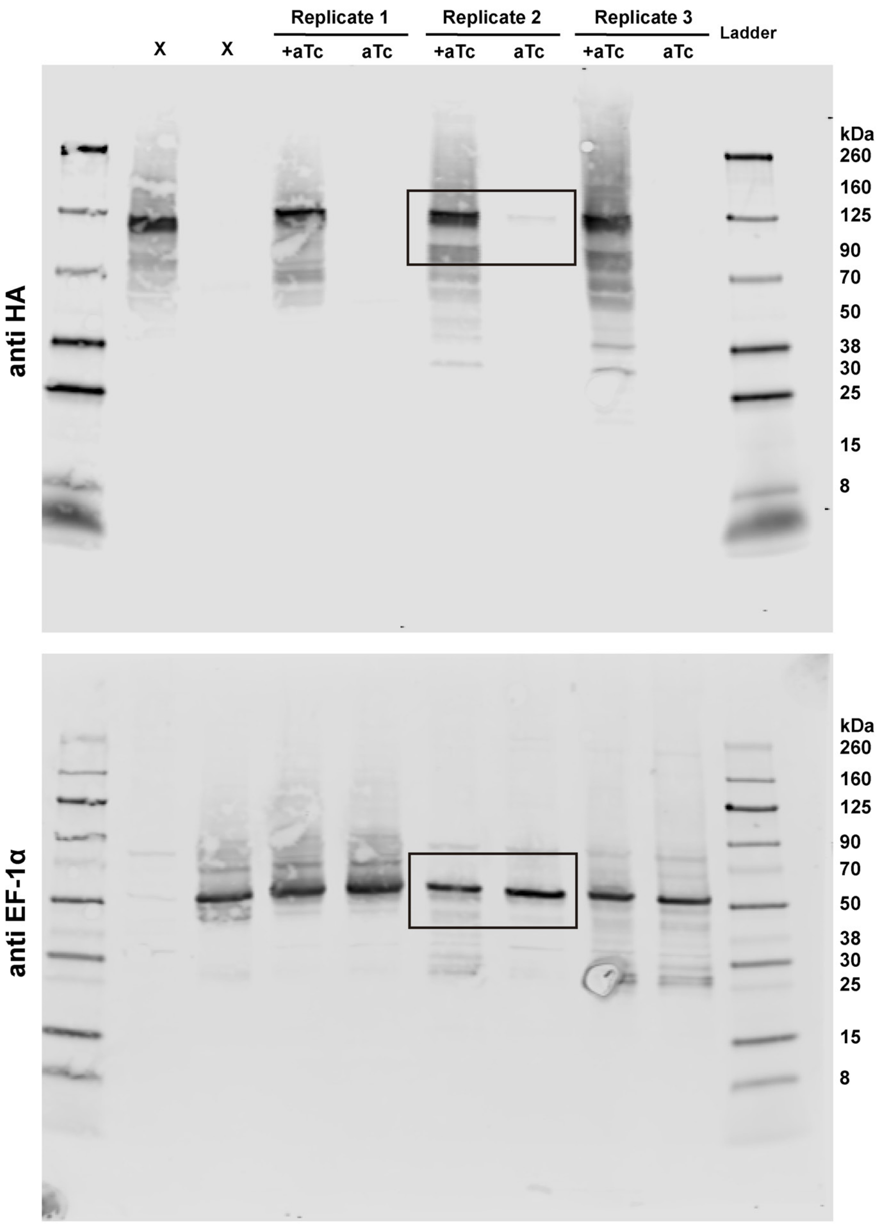
Replicate 1
Replicate 2
Replicate 3
Ladder
X
X
+aTc
aTc
+aTc
aTc
+aTc
aTc
anti HA
kDa
260
160
125
90
70
50
38
30
25
15
8
anti EF-1α
kDa
260
160
125
90
70
50
38
30
25
15
8

Figure 5B

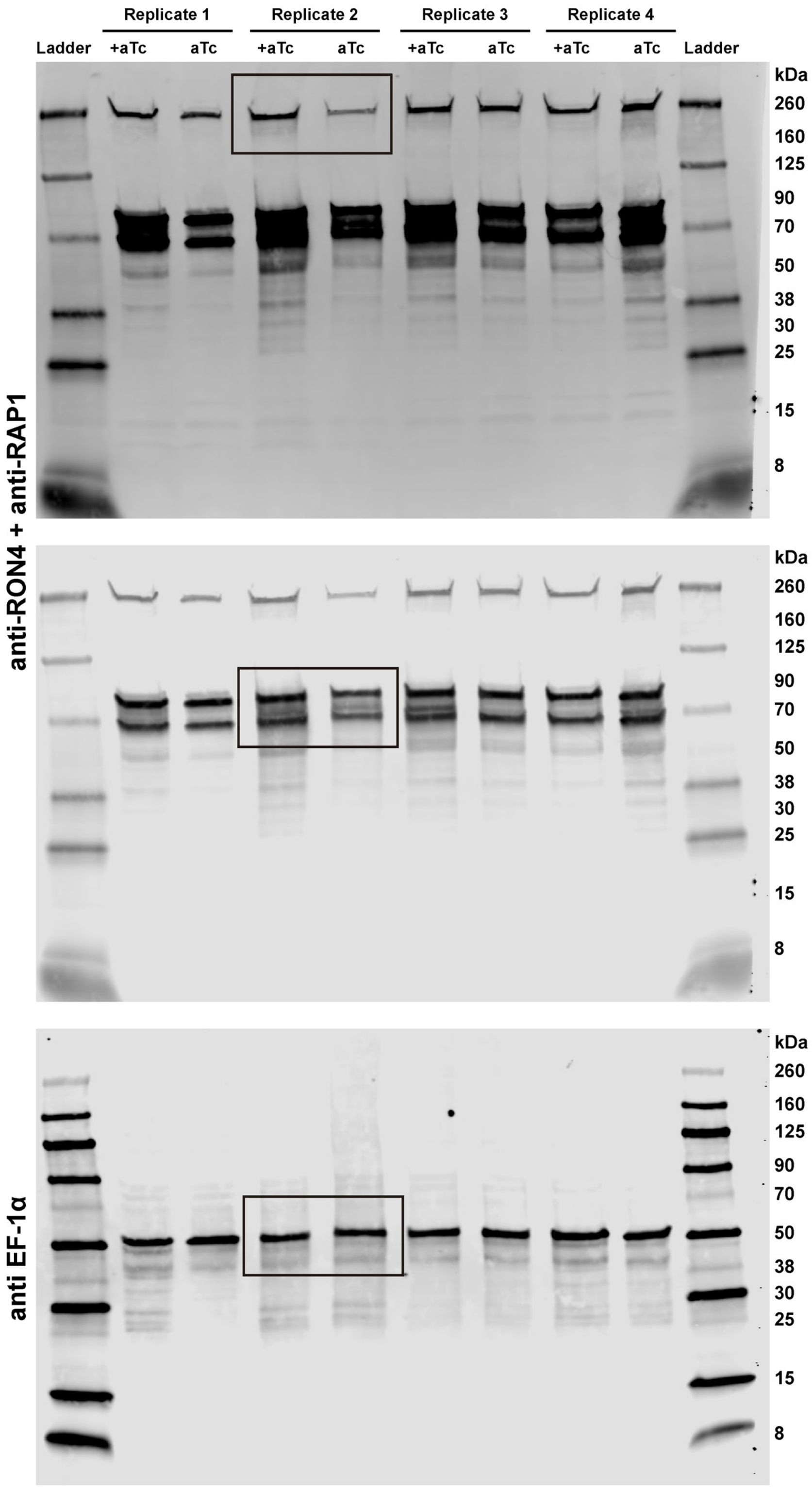
Replicate 1
Replicate 2
Replicate 3
Replicate 4
Ladder
+aTc
aTc
+aTc
aTc
+aTc
aTc
+aTc
aTc
Ladder
kDa
260
160
125
90
70
50
38
30
25
15
8
anti-RON4 + anti-RAP1
anti EF-1α

Supplementary figure 2A

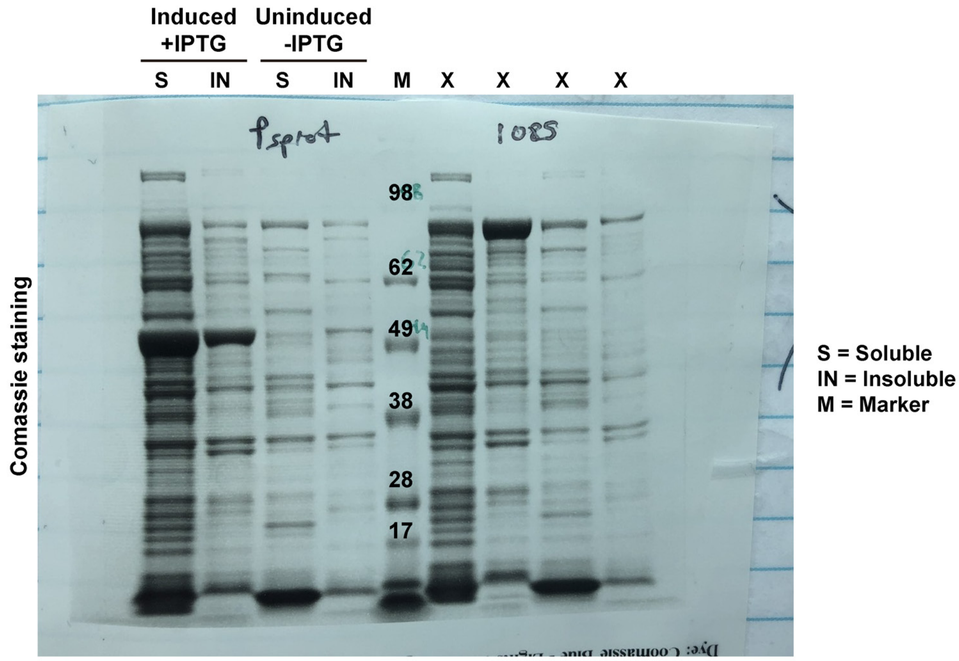

Supplementary figure 2B

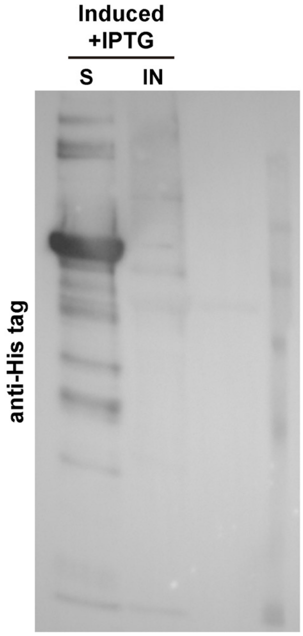

Supplementary figure 2C

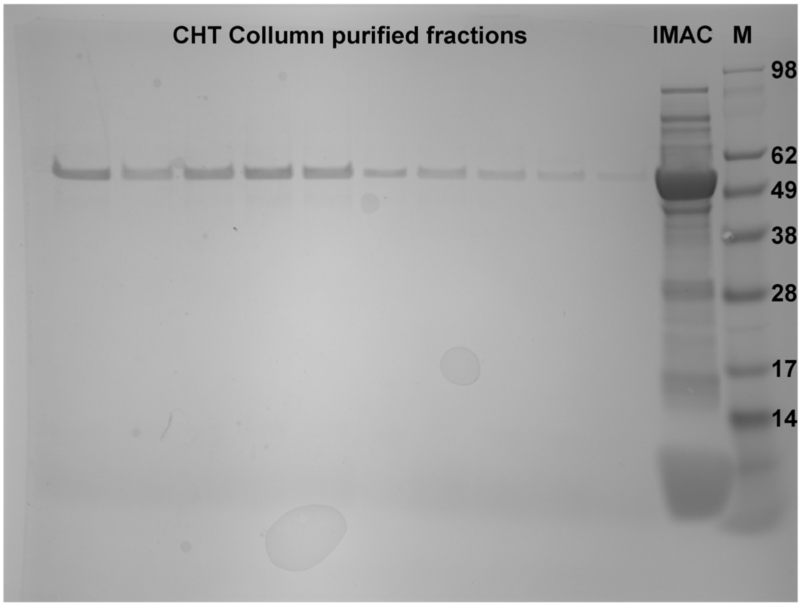

Supplementary figure 2D

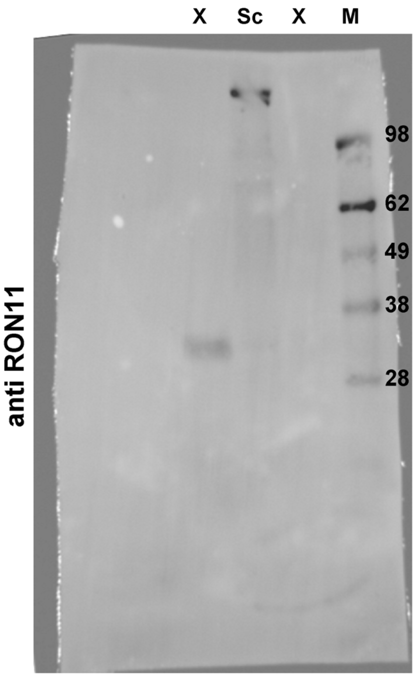

Sc = Schizont lysate
M = Marker

Supplementary figure 5B

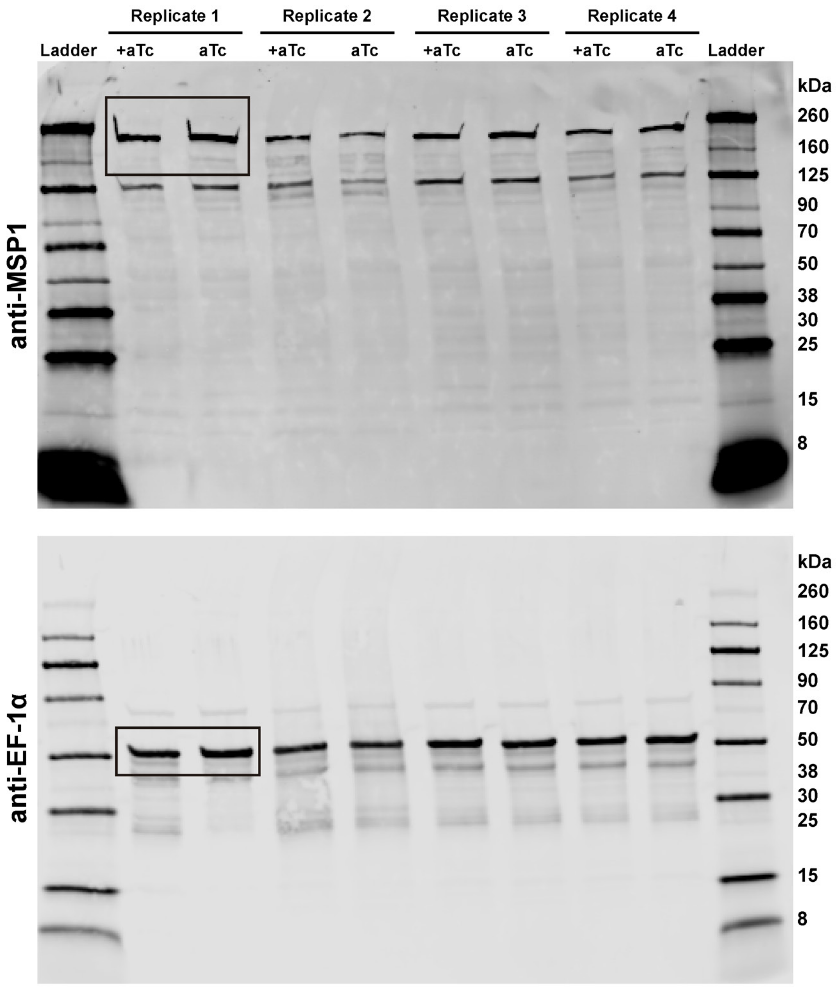

Supplement: S1 Raw Images — (PDF) [file pbio.3002801.s012.pdf]
