# Supplementary material for: Plasmodium RON11 triggers biogenesis of the merozoite rhoptry pair and is essential for erythrocyte invasion
Source: PLoS Biol. 2024 Sep 18;22(9):e3002801. doi: 10.1371/journal.pbio.3002801 (PMC11441699; doi:10.1371/journal.pbio.3002801)
Supplement: Attachment — Submitted filename: RON 11 Review Response.docx [file pbio.3002801.s013.docx]

All our responses are in red.

Reviewer #1:

This study by Anaguano et al. investigates the role of Rhoptry Neck Protein 11 (RON11) in Plasmodium falciparum, the causative agent of malaria, with a focus on its involvement in erythrocyte invasion and rhoptry biogenesis. Utilizing conditional mutants of RON11, the study reveals its indispensable nature for parasite growth, primarily through its facilitation of merozoite invasion into red blood cells (RBCs). Remarkably, the loss of RON11 results in a unique phenotype where merozoites possess only one rhoptry each, impeding successful invasion despite initial attachment to RBCs and eventual secretion of rhoptry contents. Their observation suggests a novel function for RON11 in triggering the de novo formation of the second rhoptry during merozoite segmentation, shedding light on the intricate regulation of rhoptry biogenesis. The study hints at a potential role for RON11 in regulating the synthesis and secretion of rhoptry proteins, possibly mediated through calcium signaling pathways. In my view, this study provides crucial evidence and avenues to understand, and eventually elucidate, a longstanding question of why P. falciparum merozoites need 2 separate rhoptries and only fuse them upon secretion, and whether there are differences between the two rhoptries. Further studies stimulated by this work will hopefully address these important questions.

The manuscript is well-written, and conclusions are well-supported by multifaceted experiments. The findings advance our understanding of Plasmodium invasion mechanisms and pave the way for further exploration into the biology of the important organelle, the rhoptry, in apicomplexan parasites. I would like to congratulate the authors on this outstanding piece of work.

- Response: We thank the reviewer for their exceptionally positive and constructive feedback. We are grateful for their foresight and recognition of our work. We have edited the resubmission to address their specific comments.

Specific comments:

1) Line 273: "...which is thought to be indicative of secretion of contents from rhoptries into the RBC" - needs a reference.

- Response: We added the reference.

2) I have trouble understanding the plots in Fig. 4B. The description is "Quantification of merozoites with single, dual, or multiple rhoptries in the presence and absence of aTc." Are each dot on the plot representing one merozoite? If so, what does the Y-axis "percentage of merozoites" mean for each dot?

- Response: The figure legend has been clarified. Each dot represents one schizont (and the % of merozoites within the schizont with one, two or more rhoptries).

3) Line 543: "…only two are known to release their contents during invasion (Mageswaran et al. 2021)" - the referenced paper showed evidence for two rhoptries being ready for secretion at a time, but it's not concluded if only two rhoptries are secreted for invasion. Please revise.

- Response: The claim has been revised and edited.

----------------------------------------------------------------

Reviewer #2:

Red blood cell invasion of the malaria parasite is an essential, highly unique and specialized process. This tightly coordinated cell-cell interaction relies on secretory organelles including the micronemes, rhoptries and dense granules that secrete their contents to facilitate interactions. Rhoptries are the largest and most prominent of these invasive organelles. They are very likely Golgi-derived and manifest as dual-club-shaped organelles located at the apical pole of the invasive stage of the parasite. They are formed de novo during cytokinesis, are storing a subset of secreted proteins and secrete them during host cell invasion. Despite the essential role of these organelles for parasite proliferation, rhoptry biogenesis and function is not well understood.

The authors of this nice manuscript are investigating the function of a multi-transmembrane rhoptry protein termed Rhoptry Neck Protein 11 (RON11). By generating conditional RON11 mutants using the tetR-DOZI aptamer system, they show that the knockdown of RON11 interferes with efficient red blood cell invasion and therefore blocks parasite proliferation. They show that RON11 deficient merozoites are able to engage with RBC and release their rhoptry content but are prevented from a productive invasion process. The authors show that the loss of RON11 leads to a decrease in the amount of rhoptry proteins. Amazingly, this goes with a drastic morphological change of the rhoptries which are reduced to a single one. Nevertheless, this "freak" rhoptry appears to be perfectly orientated within the complex architecture of the apical tip of the parasites and apparently accommodates a set of rhoptry proteins that are normally processed. They conclude that RON11 triggers the de novo biogenesis of the second rhoptry and that is apparently necessary for RBC invasion.

This is a well-written manuscript with a thorough functional analysis of a gene coding for the multi-transmembrane rhoptry RON11 that was shown in Toxoplasma not to be essential for host cell invasion. Using a conditional knock-down system in P. falciparum the authors convincingly show that RON11 plays an important in parasite RBC invasion and present a remarkable single rhoptry phenotype.

- Response: We thank the reviewer for recognizing the importance of our study and for their positive evaluation. The resubmission has been edited (as below) to address their specific feedback.

A couple of remarks:

1.	According to the growth curve in 1D, they observed a clear growth defect only after the second cycle without aTC. It is not clear when they collected the samples to corroborate the knock-down by WB. Was it after one or two cycles without aTC? How old were the schizonts that they analyzed for invasion and microscopy experiments, do they have to keep them for two cycles without aTC to see the effect of knock-down? The information might be included somewhere but this reviewer could not find it and it appears to be important to state this upfront to allow the reader the assessment of these data points.

- Response: We have clarified this and added information in Materials and methods.

2.	Fig. 2: Please quantify parasite invasion (number of rings per ruptured schizonts- compared with control). Any egress (percentage of ruptured schizonts compared to control) phenotype?

- Response: We are unsure how to precisely calculate the number of rings/schizont rupture. A simple ratio of schizonts and rings at each time point could illustrate this point but that did not demonstrate anything different from the data in Fig. 2B (Figure here). As the data in Fig. 2B show, equal amounts of schizonts rupture after removal of compound 1. In both + and – aTc conditions, the schizont numbers reduce from ~4% to1% with almost identical kinetics. Together with thin blood smear data, these data show that there are no egress defects observed upon RON11 knockdown.

3.	The authors claim that that the N terminus of RON11 is accessible to antibodies during merozoite invasion and may interfere with its function in RBC invasion. What is the basis for this? Would be nice if this implied topology would be confirmed with some experimental data such as digitonin/ proteinase K protection assays?

- Response: The orientation is based on predicted membrane topology in the ER during protein synthesis. We agree that experimental data is needed to confirm this and future (ongoing) work will determine the exact orientation of RON11 in the rhoptry membrane. We are using a GFP-nanobody based approach to determine if the membrane topology of RON11 matches its predicted orientation. However, this work is beyond the scope of the current manuscript.

4.	The authors observed that RON11 knockdown also led to about a 50% reduction in the amount of rhoptry proteins in general - likely because of merozoites with a single rhoptry. As they claimed, this is unexpected, because a defect in organelle biogenesis should not affect protein expression. This reduction is not really fully supported from the WBs shown, although they quantified by densitometry in several experiments. They used EF1 as loading control but a loading control with a similar time of rhoptry protein expression (RON2, RAP1, AMA1, …) would be more appropriate to exclude a potential developmental delay.

- Response: A different WB has been used to illustrate the data. We agree that a protein with similar developmental expression would be appropriate, though the reduction in the amount of RON4 and RAP1 precludes their use as controls. Hence, we quantified the expression of MSP1 (which is also only expressed in schizonts) both by WB and IFA to ensure that the loss of RON11 had no effect on schizont development (Supp. Fig. 6).

5.	Fig 6. - rescued RON11 knockdown phenotype by addition of aTC: Please provide data on the morphology of their rhoptries and on their protein content.

- Response: We observed the rescued RON11 knockdown by expansion microscopy. These data show that within a single schizont, some merozoites develop with two rhoptries, while others still have a single rhoptry. These data suggest that, within a single schizont, the merozoites are not developmentally identical and hence, some are able to respond to RON11 restoration while it is too late for other merozoites.

6.	Discussion: It might be advisable that the authors summarize the data they provided and align them with the two different putative functions they hypothesise: i) "RON11 triggers the de novo biogenesis of the second rhoptry" ii) "RON11 through its C-terminal EF hand motifs, which can bind calcium, may play a role in regulating the kinetics of rhoptry secretion and/or subsequent steps in invasion. While the first suggestion is supported by the mesmerizing

phenotype, for the second function the data are sparse.

- Response: We agree that the data are sparse for an independent function of RON11 during invasion. The ability to inhibit invasion using anti-RON11 antibodies does suggest a more direct role for RON11 in merozoite invasion. While there are several ways to parse the discussion, we would like to discuss the two hypotheses together precisely because our data, at this time, cannot fully distinguish between the two. Future work will clarify if there are two separate functions or if they are linked.

7.	Just out of interest - why an HA and not GFP tag?

- Response: We were worried that a GFP tag may interfere with the C-terminal EF hand function. In our experience (and probably the wider field as well), peptide tags generally don’t interfere with function of the protein.

However, experiments are underway to tag it with GFP, since we are interested in observing RON11 localization in live parasites.

----------------------------------------------------------------

Reviewer #3:

This report details a fascinating analysis of the role of the malarial rhoptry neck protein, RON11. The protein is found across the apicomplexa and previous indirect evidence has suggested that it may play a critical role during invasion. Here the authors use a conditional knockdown approach to demonstrate that the protein is required for both rhoptry biogenesis and invasion - with a unique and very specific KD phenotype of production of merozoites with only a single rhoptry as opposed to the standard pair. The work is elegant, methodical, and very well written. The work also provides a unique and novel insight into two key areas of invasion and organellar biogenesis, which will be of quite wide interest to those interested in host pathogen interactions. My comments are relatively minor corrections to wording with a few missing controls which could hopefully be added without too much additional work.

- Response: We are grateful to the reviewer for their constructive review and for their enthusiastic evaluation of our work. Their specific comments are addressed below.

L62 - This phrasing might be read to imply that invasion as a target is in use within a clinical setting, which it is not. There is of course strong interest in developing invasion targets. Rephrase sentence accordingly, also not sure González-Sanz, Berzosa, and Norman even mention invasion targets.

- Response: We thank the reviewer for catching this mistake. We have clarified the initial claim and corrected the erroneous reference.

Line 147, a KD of 97% of protein is quoted, but there doesnt seem to be a band at all in -aTc HA land of 1C. How was the 97% calculated? If a longer exposure was used, then this one should be used for fig 1C.

- Response: We included an overexposed blot on supplemental figure 1 to clarify this observation. The expression levels were quantified by densitometry using EF-1α as a loading control.

Figure 1A schematic primers are labelled incorrectly. In the integration line I beleive it should read P1 P2 then P6 in order to match with Fig 1B (or Fig B could be changed to match 1A and the P6 in parental line changed to P2). Could the authors also confirm that P1 is situated outside of sequences used in the template plasmid? Perhaps it could be moved slightly on schematic to make this more clear (important so that P1 +P6 cant create product with episome).

- Response: We corrected the schematic to match Fig 1B, and to clarify that P1 is localized outside of the template sequence.

Figure 1 Legend - This could be edited down a bit to be more concise. Some of the others could do with a bit of a trim too.

Figure 2C should have a control IgG, either rat preimmune or a rat IgG control.

- Response: All figure legends have been trimmed. The rMBP was used as a control since the antibodies were generated using MBP-RON11 fusion protein.

Supplemental figure 2- IFA data comparing HA and RON11 antibody specificity is convincing, but also demonstrating loss of signal in ATC- cultures would have been perfect control.

- Response: We included an IFA figure in Sup Fig 2 showing lack of signal in the absence of aTc.

L270 Reorientation is not measured at any point, despite the broad assumption within the field parasites can theoreticall apically attach without reorientation, there is also no evidence to say that echinocytosis cannot happen without reorientation. I think it is important not to assume this step has taken place, so I suggest mention of reorientation is removed to stick to directly observed phenotypes.

- Response: We agree and therefore, mentions of reorientation were removed from manuscript.

L296 - I am confused by what 3D and 3E are showing. time from contact to echinocytosis and time of echinocytosis? What period is E measuring? If this is simply from start of the videos then you would instead conclude that the RON11- parasites take longer to make initial contact? Or is it the duration of the echinocytosis itself?

- Response: We apologize for the confusion. The figure and the legend have been edited. The data show the duration of echinocytosis itself.

Figure 3 legend. Colours for F do not match between figure and legend (RAP1 is listed as red in legend, but yellow in figure etc).

- Response: Thank you for catching this error. It has been corrected.

L440 "supplementation after 46 hours unable to rescue growth"? Should this not be 48 hours onwards? Could the authors clarify the lifecycle length of the parasites they are using? Most lines have a lifecycle length significantly shorter than 48 hours. It may be more logical to talk about hours prior to egress than post invasion in this context.

- Response: We are using the NF54^attB^ parasite line, which has a 48 hour asexual life cycle. We have clarified this duration in the resubmission. We have better control over invasion since we use a combination of Percoll purification and Sorbitol lysis to ensure an exact window of invasion, as described in the methods.

L584 - Can authors note whether the EF hands are likely in the luminal or cytosolic face.

- Response: Edited to include the predicted orientation of the EF hands.

Minor

L190 remove "As" - there is no follow-on statement within the sentence. Could also change showed to "have shown".

- Response: Corrected.

All the best,

Rob Moon

— — —

ADDITIONAL ACADEMIC EDITOR COMMENTS.

This well written manuscript’s dissection of the time of the invasion defect post attachment, reorientation and deformation of the red blood cell and the demonstration that RON11 is not required for rhoptry secretion are important contributions to understanding the role of RON11 in rhoptries in invasion. The ability to produce only a single rhoptry is also a novel Discovery and important contribution to understanding rhoptry biogenesis.

More detail could be provided for the methods, fig legend and description of the uexm. The authors use the shorthand of Liffner et al of “NHS-ester” for NHS-ester conjugated to a fluorescent dye that non-specifically labels proteins, this needs to be explained by the authors as it is unclear from the current MS how the use of NHS-ester stains the centriolar plaques. The evidence that the knockdown merozoites have only a single rhoptry seems clear and the expanded microscopy clearly indicates segregation of a single rhoptry but it isn’t clear to me that this is occurring after the final mitosis in the knockouts. The basal complex rings are clearly evident in the control in fig 7d but not so clear in the knockout. I agree that the authors explanation is the most likely explanation but it would be strengthened by provision of additional images as a supplement and some analysis of number of segregated single rhoptry vs double in KO and control parasites with a clear basal complex ring would also strengthen the argument.

- Response: We thank the editor for their positive evaluation of our work.
- We have included more details in the methods to clarify how we are using NHS-ester to stain centriolar plaques. Briefly, you are correct that the NHS-ester is a general protein stain and the only way we are observing CP is because it is a protein-rich complex localized near the apical polar ring. We have also included more images (Supp. Fig. 7) to show more images where the basal ring is clearly observed.
