# Supplementary material for: Plasmodium RON11 triggers biogenesis of the merozoite rhoptry pair and is essential for erythrocyte invasion
Source: PLoS Biol. 2024 Sep 18;22(9):e3002801. doi: 10.1371/journal.pbio.3002801 (PMC11441699; doi:10.1371/journal.pbio.3002801)
Supplement: Attachment — Submitted filename: Response-to-editor.docx [file pbio.3002801.s014.docx]

The authors have addressed the editor’s comments satisfactorily. They have addressed most of the reviewers’ comments with two exceptions, they have not addressed R2’s request to confirm membrane topology by enzymatic digestion as they say it is beyond the scope of the project. However R2 points out that they claim the N terminus of ron11 is an antibody target during invasion. R2 only says it would be nice to include these data so I think it is not essential, but I think they should remove any speculation about exposure to antibodies if they will not do the digestion assays.

Response: We thank the editor for their positive assessment of our revised submission. We have removed the measured speculation about exposure to antibodies (lines 180-182).

R3 asked for a non-specific antibody control for the growth inhibition assay in fig2c. The authors have not provided this data, the control provided doesn’t address the reviewers request, it only shows that the absorbable anti-rMBP portion of the IgG doesn’t inhibit growth, it does not show that the rat IgG does not have inhibitory effect. This is an easy experiment to do and the authors should have done it.

Response: We agree, therefore, we added the rat IgG control (Fig. 2C). As expected, the control IgG antibodies had no effect on merozoite invasion.

R2 also asked for the number of rings per schizont rupture to be quantified, the authors said they were unsure how to do this, their fig 2C shows that there are far fewer rings per schizont in the - RON11 than the + RON11. Their invasion assay uses percoll purified schizonts from sorbitol synchronised cultures. I think the reviewer might have been asking for magnet/percoll purified trophozoites to be invasion blocked with heparin, this generates very synchronous late stage schizonts which invade upon removal of the heparin block. This would probably be more accurate but I think the obvious phenotype in the presented data is adequate.

Response: We agree that heparin blocked parasites is another approach to isolate late-stage schizonts. However, since we isolated late-stage schizonts using PKG inhibitors, we kept this approach across all experiments. Both approaches have been utilized extensively in literature.

The authors have removed the previous fig S5 so what was s6 is now s5 and they have added a new fig s6 which addresses R2’s request for rhoptry morphology upon KD rescue. This response is sufficient but was difficult to parse, in general the authors wrote a very clear MS but their response did not make clear whether they were reiterating results that were in the original MS, or had added new data, or were just responding to comments without altering the MS. It would be a great aid to assessing their revision if they explicitly indicate exactly what they have changed and where exactly the changes are located.

Response: We apologize for this oversight in our response to reviewer comments. The data presented in new Fig. s6 are new data obtained to answer reviewer comments. The data in previous/old Fig. S5 is now part of Fig. 5 in the main manuscript.
